# Antifungal and anti-biofilm activity of a new Spanish extract of propolis against *Candida glabrata*

**DOI:** 10.1186/s12906-021-03323-0

**Published:** 2021-05-21

**Authors:** María Coronada Fernández-Calderón, Laura Hernández-González, Carolina Gómez-Navia, María Teresa Blanco-Blanco, Rosa Sánchez-Silos, Leopoldo Lucio, Ciro Pérez-Giraldo

**Affiliations:** 1 Departamento de Ciencias Biomédicas, Área de Microbiología, Facultad de Medicina y Ciencias de la Salud, Universidad de Extremadura, Badajoz, Spain; 2 Centro de Investigación Biomédica en Red de Bioingeniería, Biomateriales y Nanomedicina (CIBER BBN), Badajoz, Spain; 3 Instituto Universitario de Investigación Sanitaria de Extremadura (INUBE), Badajoz, Spain; 4 Servicio de Microbiología, Hospital Universitario de Badajoz, Badajoz, Spain; 5 Laboratorio de Salud Pública (LSP), Hospital Universitario de Badajoz, Badajoz, Spain

**Keywords:** *Candida glabrata*, Propolis, Antifungal activity, Biofilm

## Abstract

**Background:**

Resistance to traditional antifungal agents is a considerable health problem nowadays, aggravated by infectious processes related to biofilm formation, usually on implantable devices. Therefore, it is necessary to identify new antimicrobial molecules, such as natural products, to develop new therapeutic strategies to prevent and eradicate these infections. One promising product is propolis, a natural resin produced by honeybees with substances from various botanical sources, beeswax and salivary enzymes. The aim of this work was to study the effect of a new Spanish ethanolic extract of propolis (SEEP) on growth, cell surface hydrophobicity, adherence and biofilm formation of *Candida glabrata*, a yeast capable of achieving high levels of resistance to available anti-fungal agents.

**Methods:**

The antifungal activity of SEEP was evaluated in the planktonic cells of 12 clinical isolates of *C. glabrata.* The minimum inhibitory concentration (MIC) of propolis was determined by quantifying visible growth inhibition by serial plate dilutions. The minimum fungicide concentration (MFC) was evaluated as the lowest concentration of propolis that produced a 95% decrease in cfu/mL, and is presented as MFC_50_ and MFC_90_, which corresponds to the minimum concentrations at which 50 and 90% of the *C. glabrata* isolates were inhibited, respectively. Influence on cell surface hydrophobicity (CSH) was determined by the method of microbial adhesion to hydrocarbons (MATH). The propolis effect on adhesion and biofilm formation was determined in microtiter plates by measurement of optical density (OD) and metabolic activity (XTT-assay) in the presence of sub-MIC concentrations of SEEP.

**Results:**

SEEP had antifungal capacity against *C. glabrata* isolates, with a MIC_50_ of 0.2% (v/v) and an MFC_50_ of 0.4%, even in azole-resistant strains. SEEP did not have a clear effect on surface hydrophobicity and adhesion, but an inhibitory effect on biofilm formation was observed at subinhibitory concentrations (0.1 and 0.05%) with a significant decrease in biofilm metabolism.

**Conclusions:**

The novel Spanish ethanolic extract of propolis shows antifungal activity against *C. glabrata*, and decreases biofilm formation. These results suggest its possible use in the control of fungal infections associated with biofilms.

## Background

The frequency and diversity of fungal infections has changed in recent years, with a greater incidence of the opportunistic *candida* species; this is mainly due to increased immune-depressed patients, and to limitations of efficient antifungal agents [1]. *Candida glabrata* is a human commensal species which is present, among other places, in the digestive tube and with 15–50% of carriers [2–4]. Currently it is considered an emergent opportunistic pathogen in hospitalized patients, with a high incidence in *Candida* spp. isolates, behind only *Candida albicans* and sometimes *Candida parapsilosis* [5, 6]. Pathogenicity of *C. glabrata* is related with determined virulence factors such as adhesion capacity and subsequent biofilm formation [7]. There exists a direct relation between the hydrophobic characteristics of the cellular wall of the yeasts and their adherence capacity. Hydrophobicity occurs when the yeast is not capable of interacting with the water molecules, either by ion-dipole interactions or by hydrogen bonds; the degree of intervention of the hydrophobic interactions depends on the characteristics of both the fungus and the surface [8]. Adhesion is the first step in the colonization and invasion of the tissues. The adhesion capacity prevents the yeast from being dragged and allowed to develop a biofilm which protects them from adverse conditions, favours their survival, and allows access to the blood stream and to the patient’s internal organs.

Biofilms are clusters of viable and non-viable micro-organisms which accumulate in a liquid-solid interface, embedded in a mucilaginous matrix composed of polymeric extracellular substances attached to a surface [9]; the matrix provides protection for the micro-organisms through concentrations of nutrients, impeding the access of biocides, oxidants, antibiotics, metallic cations and toxins. Thus biofilms play a fundamental role in infections related with implanted medical devices such as catheters, and dental, urinary or cardiac prostheses [10]. Infections by biofilm*-*forming fungi are more difficult to eradicate and *Candida spp.* has been associated with biofilm-related infections [11]. *C. glabrata* is capable of colonizing mucous surfaces and medical biodevices, and presents resistance to frequently used antifungals [4]. There is an urgent need, therefore, to find new therapeutic strategies with possible candidate products, preferably natural, for their use in the prevention and control of this type of infections; the development of new drugs from natural compounds for control of biofilm-related infections, which present fewer side effects, is at present receiving much attention [9, 12, 13].

Propolis is a natural non-toxic product obtained from bee-hives, and is used by bees for the construction and restoration of the honeycomb [14]. It has antimicrobial, anti-inflammatory, anti-oxidant, immune-modulating and anti-tumour activity; it is therefore a product of great interest for the development of new medicines [15]. In recent years several studies have shown the antibacterial and antifungal effects of propolis, with variations according to its different origins [16–18], but few studies have been performed on clinical isolates of *C. glabrata* [19–21]. In previously published works we have verified the excellent antibacterial activity of the novel Spanish Ethanolic Extract of Propolis (SEEP) [22] and its foreseeable initial mechanism of action against gram-negative and gram-positive bacteria [23]. The chemical profile of SEEP was determined by Liquid chromatography–mass spectrometry (LC-MS) and high-resolution gas chromatography coupled with mass spectrometry (GC-MS), and revealed for the first time the presence of olive oil compounds (Vanillic acid, 1-Acetoxypinoresinol, p-HPEA-EA and 3,4-DHPEA-EDA). In addition, it should be noted that it contains high amounts of polyphenols (205 ± 34 mg GAE/g), with unusually more than half of these from the flavonoid class (127 ± 19 mg QE/g). The TFC/TPC ratio (total flavonoids content/total polyphenols contents) (~ 0.6) was the highest ever reported, both in Spain and in many parts of the world [22].

Thus, the aim of this study was to evaluate the usefulness of SEEP in the control of biodevice-associated infections caused by *C. glabrata*. To do so, we assessed its antifungal activity in clinical isolates of *C. glabrata*, and we analysed its ability to prevent biofilm formation and its effect on pathogenic yeast factors, biofilm-related, such as hydrophobicity, and adherence capacity.

## Methods

### Strains and culture conditions

Twelve *C. glabrata* strains, isolated from different patients and of different origins, seven from hemoculture (12, 20, 30, 42, 45, 52 and 62), one vaginal (64), one from sputum (63), two exudates (60, 61), and one from sample preservation liquid (65) at a tertiary care hospital (Hospital Universitario de Badajoz, Spain) were included in this study. The isolated *C. glabrata* strains were sent to us anonymized, in accordance with the protocol approved by the Ethical Committee of the Hospital, (Comité de Ética de la Investigación Clínica del CHUB), in order to maintain the confidentiality of the patients, and were included in our collection (MicromedBA). The clinical isolates were identified by sowing in chromogenic medium CHROMagar Candida® and with biochemical methods using the API Candida ID32C system (bioMèrieux, Marcy L’Étoile, France). Sensitivity to antifungals was assessed by means of the automatic system Sensititre YeastOneR (Trek Diagnostic Systems, United Kingdom). Yeasts were conserved in vials at − 80 °C (Microbanc, Prolab, Ontario, Canada). Later, the strains were cultivated on Sabouraud agar plates (OXOID LTD., Basingstoke, Hampshire, UK) and incubated at 37 °C for 24 h; two subcultures were made in RPMI-1640 (Sigma, St. Louis, MO, USA.) medium supplemented with 2% glucose, and at pH 7 in MOPS buffer (Sigma) for the different experiments.

### Propolis samples preparation

The samples of propolis employed for analysis were from a new SEEP in 70% alcohol (v/v). The propolis was collected in the southwest of Spain and provided by “La Virgen de Extremadura” (Herrera del Duque, Badajoz, Extremadura, Spain). The SEEP was filtered as received with a 0.20 μm syringe filter (Millipore, Merck, Darmstandt, Germany) and stored at 4 °C until use. This stock of SEEP, 100% (v/v) is equivalent to 61.5 g/L dry weight of propolis [22],

### Antifungal activity of SEEP on planktonic cells

In this work we studied the in vitro antifungal effect of SEEP against planktonic *C. glabrata*. For the experimental assay, serial dilutions were performed and the effect of concentrations ranging from 0.8% of SEEP (equivalent to 480 μg/mL) to 0.05% (eq. 30 μg/mL) were studied.

The minimum inhibitory concentration (MIC) of the propolis extract was determined using agar dilution method [24] on 12 *C. glabrata* strains. Activity of the extract was evaluated by determining the minimum dilution which inhibited visible growth of *C. glabrata* on the plates. The following protocol was used: Serial dilutions of propolis extract were prepared on 70% alcohol and were incorporated to RPMI-1640 medium with agar at 1.5%, to obtain concentrations from 0.8 to 0.05% with a maximum of 0.2% alcohol, and transferred to Petri plates. A standardized suspension of yeasts, prepared in RPMI-1640 at a concentration of 10^4^ cfu/ml, taken from an overnight culture, was inoculated with a drop of 10 μL, deposited on the surface. In each plate 5–7 strains were inoculated, starting with those with the lowest concentration, and left to dry at room temperature. Control plates were used, one without propolis and the other with the alcohol used as a solvent. The plates were incubated for 24 h at 37 °C and were read visually; MIC was defined as the lowest propolis concentration which produced visible inhibition of yeast growth. The results are presented as Range, MIC_50_ and MIC_90_. The MIC_50_ value corresponds to the minimum concentration that inhibits at least 50% of the strains studied. The MIC_90_ value corresponds to the minimum concentration that inhibits 90% of the *C. glabrata* strains.

The minimum fungicidal concentration (MFC) of propolis was evaluated by dilution in liquid medium in 96-well microplates, using methodology M27-A4 of the CLSI [25] with minor modifications. Briefly, 100 μl of RPMI-1640 with concentrations of SEEP of 1.6 to 0.1% (eq. 960 to 60 mg dry weight of propolis) was transferred into the 96-well microplates (Greiner Bio-One GmbH, Frickenhausen, Germany); next, 100 μl of a suspension of yeasts (approximately 10^4^ cfu/mL) was added, and final dilutions of SEEP from 0.8 to 0.05% were obtained. The microplates were incubated for 24 h at 37 °C. Subsequently, minimum fungicidal concentrations were determined according to the method of Gucwa et al.*,* [26] with minor modifications. Aliquots of 10 μl were taken from the different wells, starting from the MIC value, and were cultivated on plates of Sabouraud agar (OXOID LTD., Basingstoke, Hampshire, UK), and incubated for 24 h at 37 °C. The MFC was considered as the lowest SEEP concentration at which any colonies were observed. The results are presented as MFC_50_ and MFC_90_, these values corresponding to the minimum concentration at which 50 and 90% of the *C. glabrata* isolates were inhibited, respectively. All the evaluations were carried out in triplicate and on different days.

The inhibitory effect of SEEP on the growth kinetics of *C. glabrata* was studied [27, 28] by making a growth curve of *C. glabrata* strain 60 from shake flasks at 37 °C, with 50 ml of RPMI-1640 medium in the presence of 0.2% propolis and in control cultures without propolis and with 0.2% ethanol as solvent. The inoculum was prepared from a previous culture in RPMI-1640. Samples were taken every 2 h; their OD was measured at 492 nm until the stationary phase was reached, finalizing at 80 h of incubation. The growth specific constant (μ) was measured according to the average slope during the exponential phase.

### Effect of propolis on virulence factors of *C. glabrata*

The effect of SEEP on biofilm-forming capacity of *C. glabrata* was evaluated, as well as its effect on certain virulence factors such as adherence capacity and cell surface hydrophobicity (CSH).

#### Influence on biofilm-forming capacity

The effect of SEEP on biofilm-formation of *C. glabrata* in the presence of subinhibitory concentrations of propolis was studied (0.1 and 0.05%). Flat-bottomed 96-well polystyrene microtiter plates (Greiner Bio-One GmbH, Frickenhausen, Germany) were used for this, following the method of Ramage et al. [29] with modifications [30]. Briefly, wells of plates were filled with 100 μl of RPMI-1640 and SEEP, in quadruplicate, so that by adding 100 μl of the inoculum to each well (10^6^ cfu/ml in RPMI-1640 medium), the concentrations of propolis to be studied were obtained (0.1, 0.05%). The microtiter plates were incubated for 24 h at 37 °C with shaking, and then the plates were washed twice with cold PBS to remove the planktonic cells. The biofilm formed was evaluated by quantifying its mitochondrial metabolic activity, using the colorimetric method by reduction of XTT (Sigma, St. Louis, MO, USA.) described by Ramage et al. [29] . Aliquots of 100 μl of XTT-menadione (0,5 g/l XTT, 1 μM menadione) were added to the wells once washed, incubated for 1 h in the dark, and the colour formed was read at 490 nm. Replicates were used to obtain representative images in the inverted microscope Leica DMi8 (Leica Microsystems GmbH, Wetzlar, Germany.). A control was made without propolis and another with the solvent. Each experiment was carried out in quadruplicate and repeated on three different times.

#### Adherence capacity

Adherence capacity to polystyrene was evaluated according to Christensen’s technique with modifications, described previously by Galán-Ladero et al. [31]. Standardized suspension of yeasts with OD_492_ of 0.4 in PBS, were placed in 96-well (0.25 ml/well) polystyrene flat-bottomed microtiter plates (Greiner bio-one GmbH, Frickenhausen, Germany) in quadruplicate. Previously the yeast had been incubated 24 h at 37 °C in RPMI-1640 in the presence of subinhibitory concentrations of SEEP (0.1, 0.05%); after several washes, suspensions were prepared and the microtiter plates filled. After 24 h at 37 °C in a humid atmosphere with shaking, the suspensions were then aspirated and washed twice with cold PBS to remove non-adhering cells. A control was made without propolis and another with the solvent. The adhered layer of the wells was determined by measuring the OD at 492 nm on Universal Microplate Reader (ELx800; Bio-Tek Instruments, Inc. Winooski, VT, USA). It was considered adherent when an OD ≥ 0.05 was obtained.

#### Cell surface hydrophobicity (CSH)

The effect of SEEP on the CSH of *C. glabrata* strains was studied after incubation of the yeasts in RPMI 1640 in the presence of subinhibitory concentrations of propolis (0.1 and 0.05%) for 24 h at 37 °C. CSH levels were measured by the microbial adhesion to hydrocarbons (MATH) test, using a modification of Rosenberg’s biphasic water-hydrocarbon method [32] described by Galán-Ladero et al. [31]. Concretely, the yeast cells grown in tubes with 5 ml of RPMI-1640 with shaking for 24 h at 37 °C were harvested and washed twice with PBS 0.15 M pH 7.2 cold PBS. The washed yeasts were resuspended in PBS until an OD of 0.4 to 492 nm. To each 3 ml of suspension, 1 ml of xylene (Panreac Química SAU, Barcelona, Spain.) was added, mixed by vortexing for 1 min, and incubated until the complete separation of the two phases occurred. Two controls were made without propolis and with the solvent. The absorbance (OD) of the aqueous phase was measured and the CHS was expressed as the percentage of reduction of the initial turbidity of the aqueous phase, and classified in accordance with the criteria described by Galán-Ladero et al. [31]: < 25% low; 25–50% medium; > 50% high hydrophobicity. All the experiments were carried out in triplicate.

### Statistical methods

All data were obtained from three independent experiments at least in duplicate for each strain. The results of propolis activity on adherence, CSH and biofilm formation were processed statistically by analysis of variance (one-way ANOVA) with the statistical program SPSS v22 (Chicago, Illinois, USA). Once the samples were found to be normal (Shapiro-wilk normality test), Dunnett’s T3 test was performed if the variances were different, or by the HSD test if they were the same. Significant differences (*p* < 0.05) when compared with the control group were determined.

## Results

### Antifungal activity of propolis in planktonic cells

The initial characterisation of the strains was carried out according to susceptibility to conventional antifungal agents. A high percentage of the 12 strains of *C. glabrata* presented resistance or were sensitive dose-dependent (SDD) to fluconazol with an MIC_50_ of 32 μg/mL; most were sensitive to voriconazol (10/12, MC_50_ 0.5 μg/mL), and to caspofungin and amphotericin B (11/12, MIC_50_ 0.06 μg/mL). Two strains isolated from haemoculture, strains 12 and 30, were resistant to voriconazol, strain 30 also being resistant to caspofungin and amphotericin B (Table 1). Then, antifungal activity of propolis on planktonic cells of *C. glabrata* was studied. MICs were evaluated by the technique of dilution in agar, which turned out to be a good method since the turbidity of the propolis dilutions made turbidimetric readings difficult in a liquid medium. The data relating to the MICs of SEEP are shown in Table 1, and include ranges and concentrations which inhibited 50% of the strains (MIC_50_) and 90% of the strains (MIC_90_).

**Table 1:** Susceptibility of *C. glabrata* strains to several antifungal agents evaluated by Sensititre YeastOne and to propolis evaluated by agar dilution (MIC in μg/mL)

Antifungal Agents	Range	MIC_**50**_	MIC_**90**_
Fluconazole	1–256	32	128
Voriconazole	0.06–2.00	0.5	2.00
Caspofungin	0.06–0.250	0.06	0.125
Amphotericin B	0.25–2.00	1.00	2.00
**Propolis**	60–240	120	120

**MIC**_**50**_**:** minimum concentration that inhibits 50% of the strains

**MIC**_**90**_**:** minimum concentration that inhibits 90% of the strains

All the twelve strains of *C. glabrata* studied were very sensitive in vitro to SEEP. The propolis extract had good antifungal activity, with MIC values within the range of 0.1–0.4% (60–240 μg/mL), with a MIC_50_ and a MIC_90_ of 0.2% (120 μg/mL). SEEP exhibited excellent fungicidal activity against isolates of *C. glabrata*, with an MFC_50_ of 0.4% (240 μg/mL), an MFC_90_ of 0.8% (480 μg/mL) and range 0.4 - > 1.5%. Strain 30 isolated from haemoculture was multi-resistant to antifungal agents (fluconazole, voriconazole, amphotericin B and caspofungin), and was sensitive to propolis, with a MIC of 0.2% (120 μg/mL) and an MFC of 0.8% (480 μg/mL). Strain 12, which was isolated from haemoculture and was resistant to the azoles tested, presented fungistatic but not fungicidal activity at the concentrations studied (MIC of 0.2% and MFC > 1.5%).

**Fig. 1:**
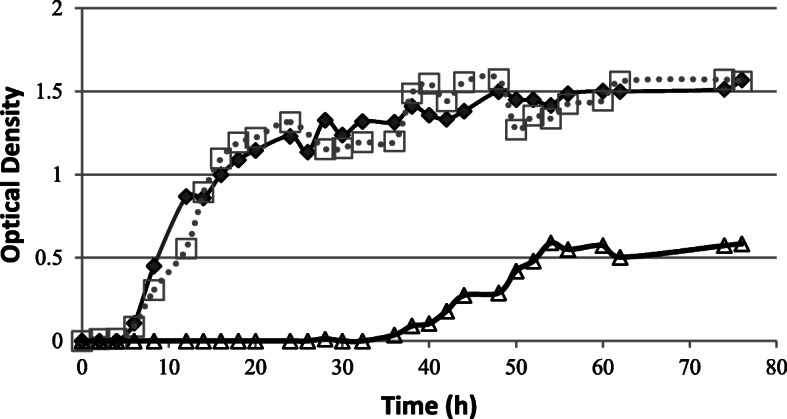
Kinetics of growth of *C. glabrata* strain 60, in RPMI-1640 medium with a concentration of propolis of 0.2% (△), and controls free of propolis ( ♦) and with 0.2% solvent (□). Optical Density of culture during 80 h of incubation at 35 °C in flask with shaking

Growth kinetics of *C. glabrata* was studied in strain 60, isolated from exudates. A growth curve in RPMI-1640 medium, according to OD values, in the presence of SEEP at 0.2% was studied. Flasks with 50 ml of RPMI-1640 at 0.2% propolis, were inoculated with 0.5 ml of yeast grown in RPMI-1640 from 24 h subculture. Growth curves in the absence of propolis and with 0.2% ethanol as solvent control were made. The growth curve with lag phase, exponential growth phase and stationary phase of the *C. glabrata* strain 60 is observed in Fig. 1. The presence of 0.2% of propolis produces a lag phase of 32 h, as observed in Fig. 1, which is much higher than the 5 h lag phase presented by the control free of propolis and the control with solvent. From this point on, the exponential phase occurs with a μ of 0.03 h^− 1^, lower than that of the two controls, without propolis and the solvent control, which was 0.09 h^− 1^ (R^2^ = 0.98), thus demonstrating the inhibitory effect in propolis growth at 0.2% (*P* < 0.05). The stationary phase was reached after 54 h of culture with a maximum biomass (0.58 OD) much lower than that of the controls (1.56 OD). The control free of propolis and the solvent control had similar behaviour in all the phases.

### Effects of propolis on *C. glabrata* virulence factors

The effect of SEEP at subinhibitory concentrations (0.05 and 0.1%) on CSH, adherence capacity, and biofilm-formation is shown in Table 2. The CSH of the yeast was measured during the early stationary phase of growth. The CSH of the strains was classified according to the criteria described by Galán-Ladero et al. [31] with the following rankings: < 25%, low; 25–50%, medium, and > 50%, high. All *C. glabrata* isolates were hydrophobic, with average CSH of 66.0 ± 27. However, the strains studied were divided into two groups, 7 strains being highly hydrophobic (> 50%) with a mean CSH value of 85.9% ± 12.4, and 5 strains presented a medium hydrophobicity with values of 38,.0% ± 7,3). No modifications produced by propolis were observed for any of the groups in CSH. The CSH of the strains increased in the solvent control (69.97% ± 29) compared to the propolis-free control (66.0 ± 27), which had a value similar to that obtained in the presence of 0.05% of propolis (71.01 ± 25). The presence of 0.1% SEEP reduced to 63.9% the mean CSH value of the strains, although the decrease was not statistically significant (*P* > 0.5).

**Table 2:** Effect of propolis at subinhibitory concentrations (0.05 and 0.1%) on CSH, adherence capacity and biofilm formation of *C. glabrata* (*n* = 12)

***Candida glabrata*** strains	CSH (%)			Adherence (OD)			Biofilm (XTT-OD)		
High CSH (Origin)	Control	0.05%	0.10%	Control	0.05%	0.10%	Control	0.05%	0.10%
12 (H)	89.2	80.3	64.5	0.09	0.10	0.10	0.38	0.31	0.29
20 (H)	89.7	80.6	55.7	0.15	0.11	0.10	0.70	0.55	0.45
45 (H)	96.0	97.3	96.2	0.13	0.10	0.11	0.61	0.40	0.31
60 (E)	91.6	100.0	100.0	0.13	0.10	0.10	0.65	0.51	0,50
61 (E)	79.3	96.2	97.7	0.12	0.10	0.09	–	–	–
63 (S)	60.7	73.7	63.2	0.13	0.06	0.08	0.50	0.40	0.31
65 (P)	94.8	93.7	83.3	0.13	0.13	0.08	–	–	–
**Median ± SD**	**85.9 ± 12.4**	**88.8 ± 10.4**	**80**.**1 ± 18**.**7**	**0.13 ± 0.02**	**0.10 ± 0.02**	**0.09 ± 0.01**	**0.57 ± 0.13**	**0.43 ± 0.10**	**0.37 ± 0.10**
**Medium CSH (Origin)**	**Control**	**0.05%**	**0.10%**	**Control**	**0.05%**	**0.10%**	**Control**	**0.05%**	**0.10%**
30 (H)	45.2	41.7	40.0	0.14	0.10	0.11	0.71	0.51	0.40
42 (H)	36.9	42.6	23.0	0.12	0.24	0.17	0.60	0.36	0.34
52 (H)	41.5	36.5	53.0	0.14	0.09	0.09	0.56	0.39	0.38
62 (H)	40.4	73.1	47.3	0.11	0.10	0.10	0.70	0.40	0.38
64 (V)	26.2	36.4	42.6	0.15	0.10	0.10	–	–	–
**Median ± SD**	**38.0 ± 7.3**	**46.1 ± 16.2**	**41.2 ± 11.3**	**0.13 ± 0.02**	**0.13 ± 0.06**	**0.11 ± 0.03**	**0.64 ± 0.07**	0.42 ± 0.07*	0.38 ± 0.03*
**GLOBAL Median ± SD**	**66.0 ±** 27	**71.0 ±** 25	**63.9 ±** 25	**0.13 ±** 0.02	**0.11 ±** 0.04	**0.10 ±** 0.02	**0.60 ±** 0.11	**0.43 ±** 0.08	**0.37 ±** 0.07*

*H* Hemoculture; *E* Exudate; *S* Sputum; *V* Vaginal; *P* Preservation liquid; (−): No data; (*) *p* < 0.05

Adhesion of yeast cells to polystyrene surface was evaluated by resuspending the cells in PBS. All the strains were highly adherent with an average OD of 0.13 ± 0.02 for both groups. Adherence capacity of *C. glabrata* incubated in the presence of subinhibitory concentrations of SEEP (0.10 and 0.05%) was not modified significantly with respect to the propolis-free control.

**Fig. 2:**
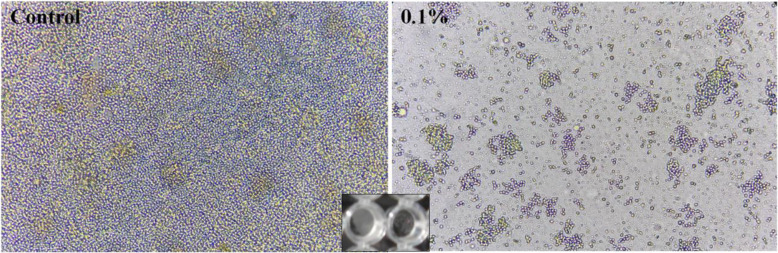
Representative images of untreated (control) and SEEP-treated (0.1%) of *C. glabrata* biofilm with inverted optical microscope (× 40)

The *C. glabrata* biofilms were quantified at 24 h of culture in RPMI 1640 by XTT reduction. All the strains were biofilm*-*forming, with an average of 0.60 ± 0.11 (range 0.38 to 0.71). Biofilm-formation diminished with the different concentrations of propolis tested in a dose-dependent way; it was observed that as the concentrations increased, metabolic biofilm activity measured by XTT decreased, compared to controls without propolis and solvent control. The presence of 0.05% propolis diminished the biofilm-formation to 0.43 ± 0.08 (range 0.31 to 0.55), and with 0.10% propolis to 0.37 ± 0.07 (range 0.29 to 0.45) in a statistically significant manner (R^2^ = 0.80; *P* < 0.05), with a more pronounced decrease in the group of moderately hydrophobic strains. This XTT-measured reduction indicates that propolis inhibits biofilm-formation. As an example of biofilm reduction, Fig. 2 shows a representative biofilm generated by *C. glabrata* strain 30 compared to biofilm after treatment with 01% of SEEP.

## Discussion

The incidence of infections by *C. glabrata* has risen in recent years due to increased use of fluconazole, and to an increase in the susceptible population, since this species is currently considered as an emergent pathogen. Drug-resistance ability of *C. glabrata* has been attributed to its haploid genome which permits it to undergo rapid genetic changes [33]. *C. glabrata* develops resistance during treatment or prophylaxis with fluconazole [7], and frequently acquires high level resistance crossed with triazoles, and also to equinocandines [34]. In the present study a high percentage of *C. glabrata* was resistant or had reduced susceptibility to fluconazole. Two strains presented crossed resistance with voriconazole, and one strain was resistant also to caspofungin and to amphotericin B, antifungals to which the rest of the strains were sensitive. Resistance to traditional antifungals is becoming an important health problem, and is aggravated in infectious processes related to biofilm-formation. There is a need to find therapeutic strategies by investigating existing products in natural environments which may have usefulness as therapeutic agents by inhibiting certain virulence factors [13, 35].

Concordant results exist on the fungistatic and fungicidal activity of propolis, although with variations in the findings related to the origin of the propolis, to the type of extract obtained and to the reading method used. Table 3 summarizes the antifungal activity of SEEP and other ethanolic extracts of propolis of different origins against *C. glabrata.* Only extracts from France [16] and Brazil [17] are reported to have a higher activity than SEEP. In relation to the methodology employed, the agar dilution method used in this work for MIC evaluation permitted very reliable readings by eliminating the turbidity produced by the propolis extract. Authors who have performed comparative studies have reported a good correlation with the CLSI reference method by microdilution [36, 37]. Some authors such as Sweda et al. [21] and Gucwa et al. [26] who used the microdilution method in broth (Table 3), did not read the MIC by propolis sedimentation, obtaining only the MFC values.

**Table 3:** Antifungal activity of ethanolic extract of propolis from different geographical origins against *Candida glabrata* strains

Candida glabrata (n)	Propolis origin	Range MIC (μg/ml or %v/v)	Range MFC (μg/ml or %v/v)	MIC Methodology	References
12	Spain	60–240 (0.1–0.4%)	240–480 (0.4–0.8%)	Agar dilution ^a^	Present work
1	France ^*^	15.63–31.25	–	Microdilution in broth ^b^	Boisard et al. [16]
14	Poland ^*^	–	1.25- > 5%		Szweda et al. [21]
1	Poland ^*^	–	0.31- > 2.5%		Gucwa et al. [26]
14	Iran	250–5000	250–8000	Microdilution in broth ^b^	Shokri et al. [19]
1	Brazil	64	256	Microdilution in broth ^a^	Siqueira et al. [20]
1	Brazil ^*^	7.8	250	Microdilution in broth ^a^	Freires et al. [17]

*MIC* minimum inhibitory concentration; *MFC* minimum fungicidal concentration by microdilution in broth and subculture on agar in all studies; (^*^) several propolis samples; (−) No data; (^a^) 100% inhibition of growth; (^b^) 80% reduction of growth

The growth kinetics of *C. glabrata* in the presence of 0.2% (120 μg/mL) shows a long lag phase. There is a significant decrease in the exponential phase growth rate and a very low biomass yield in the stationary phase. SEEP, at the MIC dose, produced a strong inhibition of the planktonic growth of *C. glabrata.*

Antifungal capacity of propolis extract in vitro is influenced by its chemical variation, due to the geographical location of the collection site and to the diverse plant origins of propolis, as has been demonstrated in several studies [18, 38, 39]. The main components of propolis are flavonoids, fatty acids and aromatic acids [40]. This new SEEP contains high amounts of polyphenols and a detailed analysis of its chemical composition revealed the presence of compounds found in commercial virgin olive oils such as Ferulic acid, Vanillic acid, 1-Acetoxypinoresinol (lignan), p-HPEA-EA (ligstroside derivative) and 3,4-DHPEA-EA (oleuperin derivative), and Vanillin (phenolic aldehyde) [41, 42]. Notably, Vanillic acid, 1-Acetoxypinoresinol, p-HPEA-EA and 3,4-DHPEA-EDA have never been detected before in propolis samples.

Several studies have attributed the antimicrobial activity of propolis to the presence of specific flavonoids and phenolic acids, such as galangin, pinocembrin, pinostrobin as well as ferulic and caffeic acids [43–45]. However, a recent study has shown that the SEEP used in this study does not contain any of the above-mentioned flavonoid agents (except for ferulic acid), and yet show similar or higher antimicrobial activity than the propolis samples that contain them. In particular, newly identified compounds in SEEP (i.e. Vanillic acid, 1-Acetoxypinoresinol, p-HPEA-EA and 3,4-DHPEA-EDA), also present in olive oil and known to possess antimicrobial properties, were proposed to be potentially responsible for the detected antifugicidal properties of SEEP [22]. In addition, it has been previously suggested that the antifungal activity of propolis is associated with the presence of the isoflavone formononetin [46], a compound that is also present in SEEP [22]. Similarly, these olive oil phenolic compounds may also contribute with their protective action in diverse diseases to healthy organisms [47, 48]. Specifically, these compounds may have protective effects against infectious, neurodegenerative or cardiovascular diseases, amongst others. Additionally, relatively high amounts of ferulic acid and quercetin were distinguished, both known for their important therapeutic benefits [49, 50].

Pippi et al. [51] carried out a comparative study of the effect of benzophenone enriched fraction obtained from Brazilian red propolis (BZP-BRP) on strains of *C. glabrata* resistant to traditional antifungal agents, and found that the strains resistant to fluconazole had good sensitivity to propolis. In our study, we obtained a similar result; the *C. glabrata* strains studied had low sensitivity to fluconazole, and were sensitive to propolis. The multi-resistant strain was sensitive to propolis extract, and only strain 12, resistant to fluconazole and voriconazole, showed greater resistance to propolis than the rest of the strains.

Most strains of *C. glabrata* have a marked hydrophobic character and high levels of adherence. Tomičić et al. [52] studied CSH with a similar protocol to that used in our study (MATH) and evaluated adherence capacity on microplates, performing the reading by staining with crystal violet. Although it is difficult to compare results when there is no standardization of protocols, there are coincident findings. CSH and adherence are closely related, but linearity between them was not obtained due to the saturation that exists in adherence capacity, as can be observed in our previous study on *Candida tropicalis* [31]. Based on the results obtained, the effect on CHS is not clear. The two resistant strains had different characteristics: both had been isolated from blood cultures but with respect to some virulence factors, they did not show uniformity. Multi-resistant strain 30 had medium levels of CSH and formed a large biofilm, while strain 12 was highly hydrophobic with a lower degree of biofilm production. This data, in accordance with previous studies, indicates that CSH does not correlate with the production of biofilms [31]. There is no evident tendency of what the effect of propolis is on the yeast surface, and in this sense, even though the subinhibitory concentrations seem to reduce adhesion of the yeast to polystyrene, it is not statistically significant.

Biofilm-forming capacity is an important resistance factor of *C. glabrata* since it contributes to the persistence of the microorganism inside the host. Biofilm formation has important clinical repercussions, and it begins with adherence to a substrate, which is related to CSH [8, 31]. The strains of *C. glabrata* studied are biofilm-forming but at a lower level than other species with filament capacity such as *C. albicans* and *C. tropicalis* [31]. Kucharíková et al. [53], in their studies on biofilm formation of *C. glabrata* using a method similar to the one used in our study, i.e. XTT reduction in 96-well polystyrene microplates and in RPMI-1640 at pH 7, obtained similar findings to ours in the control. Currently, there is widespread interest in the elucidation of matrix components, their function and their modulation by the host environment to unveil their role in the pathogenesis of *C. glabrata* [54]. These findings are crucial for the development of new therapies to prevent or eradicate *C. glabrata* biofilms. Inhibition of biofilm-formation of *C. glabrata* in the presence of propolis has been scarcely studied. In our work, we found that subinhibitory concentrations of propolis extract significantly reduce the biofilm formed by *C. glabrata* in a dose- dependent manner, without affecting other virulence-related factors such as CSH or adherence capacity.

Although the antifungal activity of propolis has been widely studied, the mechanism of action on eukaryotic cells remains unclear. Authors such as Gucwa et al. [26] suggest that the cell wall cannot be considered the target of an ethanolic extract of propolis from Poland, indicating the fungal cell membrane as the most probable site of action. In our study, biofilm formation was significantly inhibited by SEEP in a similar way to the inhibition produced in the growth of planktonic cells, while adhesion capacity and CSH were not significantly affected. This suggests, according to Gucwa et al. [26], that propolis affects yeast without impacting the cell wall, which is responsible for its hydrophobic nature and directly related to adherence capacity. Conversely, propolis affects biofilm formation by inhibiting yeast growth. This is also corroborated by the effectiveness of propolis against azole-resistant strains [19–21, 26], which act by altering the cell wall. If the antifungal compounds of propolis do not target the cell wall, the mechanisms of azole resistance do not influence their effectiveness.

Other authors such as Okińczyc et al. studied the effect of a Nepalese propolis on *C. albicans* filamentation and oxidative stress [55]. Propolis inhibited filamentation, causing a reduction in virulence because the cells became less hydrophobic. In our study, the CSH of *C. glabrata* was not affected, probably because they do not filament and unlike *C. albicans*, their yeast-like form is hydrophobic. Furthermore, they observed that propolis caused oxidative stress through the production of the superoxide anion radical (O_2_^•-^) and a lower concentration of the hydroxyl radical (OH^•^), suggesting that indirect generation of reactive oxygen species (ROS) may be the result of different processes such as disruption cell membranes.

In a previous work on the physicochemical modifications of the cell surface of bacteria by SEEP [23], we proposed that the mechanism of action of propolis against bacteria appears to be initially physical, with structural damage to the bacterial cell membrane/wall. This is a mechanism of action to which microorganisms may find it difficult to generate resistance, especially if different propolis molecules interact synergistically. Further research is needed in order to clarify the mechanism of the antifungal and antibiofilm activity of propolis in yeast. The rise in infections by *C. glabrata* and its resistance to antifungals, in particular azoles, and the fact that a large number of recalcitrant infections are related with biofilm production, point to the need for research studies to develop alternative therapies with natural products as propolis which complement traditional treatments.

## Conclusion

The novel Spanish ethanolic extract of propolis demonstrated a capacity against *C. glabrata.* In addition to its antifungal potential, SEEP reduced biofilm formation of this emergent opportunistic pathogen. This anti-biofilm activity makes it an interesting therapeutic alternative in the prevention and control of biodevice-associated infections. Future research with biomaterial coatings with SEEP could be explored to strengthen their possible use on prostheses.

## Data Availability

The datasets used and analyzed during the current study are available from the corresponding author on reasonable request.

## Abbreviations

CFU/mL: Colony forming units per milliliter
CSH: Cell surface hydrophobicity
Eq: Equivalent
MFC: Minimum fungicidal concentration
MIC: Minimum inhibitory concentration
PBS: Phosphate-buffered saline
SEEP: Spanish ethanolic extract of propolis
XTT: 2,3-bis (2-methoxy-4-nitro-5-sulfophenyl)-5-[(phenylamino)carbonyl]-2H-tetrazolium hydroxide
